# Supplying trees in an era of environmental uncertainty: Identifying challenges faced by the forest nursery sector in Great Britain

**DOI:** 10.1016/j.landusepol.2016.07.027

**Published:** 2016-12-15

**Authors:** Richard Whittet, Joan Cottrell, Stephen Cavers, Mireia Pecurul, Richard Ennos

**Affiliations:** a Institute of Evolutionary Biology, School of Biological Sciences, Ashworth Laboratories, Charlotte Auerbach Road, Edinburgh, EH9 3FL Scotland, United Kingdom; b Centre for Ecology and Hydrology, Edinburgh, Bush Estate, Penicuik, Midlothian EH26 0QB Scotland, United Kingdom; c Forest Research, Northern Research Station, Roslin, Midlothian, EH25 9SY Scotland, United Kingdom

**Keywords:** Forest nursery, Climate change, Stakeholder survey, Great britain, Biosecurity, Seed sourcing

## Abstract

In recent years, numerous articles have addressed management strategies aimed at assisting forests to adapt to climate change. However, these seldom take into account the practical and economic implications of implementing these strategies, notably, supply of forest plants and seed. Using semi-structured interviews with practitioners involved in the plant and seed supply chain in Great Britain, we highlight a series of practical and economic bottlenecks commonly encountered in the supply of locally sourced seed and domestically produced planting stock for native woodland and hedging markets. We find that adoption of alternative seed sourcing strategies, designed specifically to account for directional climate warming, is likely to exacerbate existing problems by adding further complexity to decisions nurseries make about tree species and seed origins to produce. The lack of long-term market predictability brought about by the current configuration of forestry grants and regulations and, in particular, the administrative systems for processing grant applications is identified as a major impediment to having a sustainable and competitive supply of home-grown and currently adapted planting stock. Finally, the time and effort it takes to supply healthy plants for native woodland creation projects deserves much wider recognition throughout the industry and will be crucial if planting objectives are to be met sustainably.

## 1 Introduction

A sustainable supply of germplasm or planting material is crucial for any plant based ecological restoration project (Broadhurst et al., 2016). An abundance of research emphasises that the planting material supplied for native woodland creation and restoration should come from a seed source that is ecologically and genetically appropriate for the planting site (McKay et al., 2005, Leimu and Fischer, 2008, Vander Mijnsbrugge et al., 2010, Sgrò et al., 2011, Breed et al., 2013, Bucharova et al., 2016).

A long held view, especially in conservation science has been that the use of locally sourced seed for planting or sowing is the optimal strategy. However, with increasing recognition of global climate change, it has been proposed that in future, seed should be sourced from areas which currently experience climatic conditions expected for the planting site at some point into the future (Aitken and Whitlock, 2013, Breed et al., 2013, Prober et al., 2015).

Absent from much of this ongoing debate regarding the biological considerations affecting seed sourcing is any assessment of the practical implications of seed-sourcing strategies and the impact that seed origin specification has upon forest nursery enterprise. In this paper we explore how private businesses involved in the supply of seed and planting stock of trees and shrubs for the forestry and hedging markets are affected by policies that govern seed origin choice—using Great Britain (GB), as our study region. The situation in GB, the archipelago composed of the countries of England, Scotland and Wales, is particularly pertinent because there is currently high motivation to expand and restore native woodland in many parts of the country (Scottish Executive, 2006, Forestry Commission, 2007a, Welsh Assembly Government, 2009), and due to problems with fragmentation of woodland and unreliability of natural regeneration, this is best conducted by planting of nursery-raised tree seedlings, rather the use of less resource intensive direct seeding approaches (Willoughby et al., 2004).

To achieve our objectives we have canvassed and attempted to portray opinion on these issues from members of the domestic forest nursery sector in GB. This provides us with a sound basis of critical qualitative data which is frequently communicated verbally and via various informal online platforms, but rarely discussed in the scientific literature. This information, combined with summary data from publicly held trade records (see Section 2.6), has been synthesized to provide an account of the seed supply and forest nursery sector as it exists in practice in GB, and to explore its strengths and weaknesses.

A key practical aim of the paper is to highlight bottlenecks in the forest seed and plant supply chain, i.e. identify where various practicalities or bureaucratic protocols impose constraints on the ability for the nursery sector, and their customers to follow biologically based guidance related to seed sourcing for forest trees. We look at these issues under guidelines that were designed under the assumption of a stable climate, but also explore whether changes to existing guidance which aim to account specifically for directional climate warming (e.g. Morison et al., 2010, Forestry Commission England, 2010, Forestry Commission, 2011, Weir, 2015), will complicate or ease the current status of the supply chain.

## 2 Materials and methods

### 2.1 Background and context

For native species, seed sourcing and certification in GB involves a geographical system of seed zones comprising four regions of provenance which are subdivided into 24 seed zones of roughly similar size (Herbert et al., 1999). Apart from the special case of Scots pine (*Pinus sylvestris* L.), which has customised seed zones based on patterns of selectively neutral genetic variation (Forrest, 1980, Kinloch et al., 1986), the delineation of seed zones is identical for all native species and thus fails to take into account the possibility that patterns of adaptive variation may vary amongst different species (Rehfeldt, 1994, Vitasse et al., 2009). The purpose of the seed zones is to encourage the use of locally adapted planting stock for woodland creation, i.e. a planting scheme should use planting stock which has been raised from seed collected from within the confines of the local seed zone. The requirement to use locally sourced seed is based on the premise that generations of natural selection in similar environments will have produced phenotypes best able to cope with biotic and abiotic conditions of the planting site. Using locally sourced seeds is often a requirement to obtain subsidy support for a planting scheme. However, it is worth noting that delineation of the seed zones, in their current form was somewhat arbitrary, based on major geographical boundaries and watersheds but not based on evidence of phenotypic or genetic variation in tree populations (reviewed in Whittet et al., in press).

Seed collections from native trees are typically organised by nurseries and seed merchants and conducted by contractors from wild tree populations (Herbert et al., 1999). British tree seed collection guidelines for native species suggest that collections should be made from at least 20–30, well-spaced, open-pollinated individuals which are isolated from non-indigenous stands of the same or closely related species, and should avoid selecting trees based on any particular morphological characteristics (Herbert et al., 1999). However, it is worth noting that following these guidelines is typically at the discretion of the seed collector. In addition to seed collection, seed for most species tend to require cleaning and stratification (breaking dormancy) before they are sown, which, along with seed storage, is considered by most nurseries to be a specialist activity and therefore often tends to be conducted by specialist seed merchants rather than by nurseries.

Most tree planting schemes are eligible for subsidy support via contributions from the United Kingdom’s allowance of funding from the Common Agricultural Policy of the European Union (EU). As part of the grant application process, applicants must demonstrate that the proposed planting scheme complies with regional priorities. Usually, stating the intended seed origin of planting stock is required and often the authority overseeing the proposal stipulates that seed from the local seed zone is used. However, the extent to which seed origin choice influences approval of a planting scheme can vary depending on the region and the objectives of planting. The applicant must also state the year in which they will claim for grant money following completion of work, meaning that funding is recouped once work has been successfully completed. Stating the claim year takes place before they know whether the proposed scheme will be approved, a process which involves many other protocols and assurances (e.g. Environmental Impact Assessment) and as such may take some time.

To meet demand, nurseries can trade amongst themselves, provided that EU regulations pertaining to the marketing of seeds, plants and parts of plants, collectively known as ‘forest reproductive material’ (FRM) are followed (Forestry Commission, 2007b). This may involve purchasing FRM from large scale enterprises on the European mainland which speculatively buy and raise GB provenance seed to be raised into plants for the GB marketplace (Russell and Evans, 2003). Imported plant material has been strongly implicated as a major pathway for transfer of plant pests and pathogens into the UK (Brasier, 2008) and elsewhere (Liebhold et al., 2012, Jung et al., 2015).

### 2.2 Selection of informants

Selection of informants was based on a list of 149 registered suppliers of FRM, maintained by Forestry Commission GB; the national forestry authority in GB. With expert opinion from key informants (representatives of the Forestry Commission who are in regular correspondence with the nursery sector), 34 businesses were contacted by email and invited to participate. This sample was subjectively considered to be a representative cross-section of the industry at the time as it contained nurseries of varying size, product specialities and with representation throughout all parts of GB. Of these 34, 19 responded positively. Ultimately, 14 private sector nurseries, 1 public sector nursery and 1 seed merchant were visited, based on the relevance of their business models to our questions. As a matter of convenience, we will henceforth describe all of the businesses as nurseries. Collectively, these nurseries estimated that they were responsible for the sale of approximately 83 million trees annually, although this number may include some double counting as many nurseries trade amongst each other. Nonetheless, this is likely to represent a very high proportion of the trees sold annually in GB.

### 2.3 Interviews

Interviews were conducted in person in semi-formal office settings and were held with senior staff, which always included owner/operators for sole traders and managing directors for limited companies. On three occasions, more than one interviewee was able to participate and when this was the case, the interview panel included other managerial staff.

Interviews were conducted towards the end of the lifting season (when plants are harvested for sale) in 2014, between February and April, with one interview conducted in April 2015. Interview duration ranged from 30 min to 2 h and followed a semi-structured format with a pre-defined interview guideline containing a mixture of quantitative (descriptive) and qualitative (discursive) questions (Supporting information S1**)**—although in some cases, answers were not provided, for example, most respondents were unwilling or unable to provide detailed summaries of annual sales volume by species. The interview guideline included questions on seed procurement, plant production and sales, grant schemes, attitude to climate change and open questions regarding any other bottlenecks in the supply chain and policy recommendations. All meetings were recorded digitally using a hand held voice recorder and transcribed manually.

Transcripts were analysed using a ‘grounded theory’ approach (Glaser and Strauss, 2009), which is a widely used inductive technique for qualitative research and seeks to address questions without *a priori* hypotheses or assumptions. Transcripts are coded to identify important concepts within the responses, allowing data collection and analysis to be performed simultaneously. Hypotheses are formed from the patterns which emerge in the early stages of analysis and are continually tested with repeated analysis until saturation, when no novel information emerges (Ní Dhubháin et al., 2009, Górriz-Mifsud et al., 2015).

### 2.4 Selection of quotations

Quotations reported here are used to demonstrate themes that were derived from qualitative data coded during data collection and analysis. We have aimed to provide a limited series of anonymous quotes to demonstrate the range of views held by the industry. Initially, all quotations relevant to each theme were collated and reviewed by the authors. The selection was narrowed down iteratively by the authors to maintain only those quotations which were either most pertinent to the matter at hand, or those which added important information which otherwise would be absent from the manuscript.

### 2.5 Generating nursery typologies

In order to contextualise the respondent’s views, quantitative summary data gathered from respondents were used to generate typologies of the different nurseries. Such criteria included details of size (*sales volume; number of employees*) and position in market place (*proportion of turnover generated by native species*; *dominant growth system* (containerised production or bare root production); *proportion of sales generated by own-produced versus traded stock*; *proportion of customers which were end users*). An attempt was made to apply a hierarchical cluster analysis to objectively classify the respondents into groups. However, results were difficult to interpret and not all businesses could be categorised according to these criteria (e.g. seed merchants), which would restrict the possibility of anonymising responses.

Instead, a less objective but more easily interpretable approach has been applied which is used to categorise nurseries based on three attributes. These three attributes are relative size, determined by ranking the nurseries by sales volume, as well as the number of employees and contractors; trading status, determined by self-sourced/grown versus purchased product and whether the nursery was involved predominantly in the market for exotic or native trees and seed. The latter two attributes were distinguished by using a 50% (i.e. majority) discriminator, i.e., if  >50% of the nursery’s turnover was having been derived from trade in seed or plants for native species, it has been scored as an ‘N’ for native. Otherwise, it has been scored as an ‘E’ for exotic. If the proportion is between 40 and 60% for native and exotic species, then the nursery was scored as ‘NE’. Importantly, ‘exotic’ species does not necessarily imply that the exotic planting stock is used for forestry purposes. It also includes exotic species supplied for amenity or horticultural purposes.

### 2.6 Trade records

To complement the qualitative aspect of this research, we also interrogated the Forestry Commission’s FRM databases which contain records of import and export transactions for forest reproductive material and for registration of seed collections. This national database is maintained in accordance with EU directives on trade in FRM.

## 3 Results

Grounded theory emerged as a satisfactory method of data collection and analysis for the interview transcripts. An interesting attribute of the forest nursery sector in GB is that private sector nurseries tend to be organised into professional membership groups, for example, the CONFOR nursery producer’s group (www.confor.org.uk/AboutUs/Default.aspx?pid=137) and the Horticulture Trades Association tree and hedging group (www.the-hta.org.uk/page.php?pageid=58). Members of these groups are in frequent communication and competition with one another and experience virtually identical market conditions. For this reason, common themes emerged in most interviews and hypotheses could be generated rapidly forming the basis of our results. We firstly discuss issues related to seed sourcing (Section 3.2), most notably those pertaining to procuring seed of particular seed origins for sowing in nurseries. We then discuss issues related to the next stage of the supply chain, i.e., actually supplying the planting stock to customers for planting schemes (Section 3.3). In this second section, we focus on factors identified as complicating prediction of demand (forest grant schemes and regulations) and measures taken by nurseries to counteract these difficulties (trading in live plants, contract growing).

### 3.1 Characteristics of the survey respondents

The sample was indeed found to represent a reasonably diverse set of nurseries, supporting the subjective criteria we had adopted whilst selecting informants (Table 1). Quotations used in the following sections will be accompanied with a code (Table 1) to demonstrate the category of respondent the quotation can be attributed to. These classifications are provided only to set context to the quotes and ought to be interpreted qualitatively, as there are too few respondents to make any statistical inference or comparative analysis of views held by different types of nurseries.

### 3.2 Seed sourcing

#### 3.2.1 Seed collection

Two of the nurseries surveyed, which were small producers of native species, collected seed for all of the stock they grew with only occasional exceptions. Four nurseries organised their own seed collections and typically employed contractors to do so. It is unclear what proportion of total stock produced was derived from their own collections, although collections were made for a broad suite of tree species. Other producers either did not collect at all (n = 6) or collected fairly haphazardly, when it was economically viable to do so, such as during a mast year when large quantities of seed are locally available. Other nurseries were content to rely on seed merchants as they considered that seed collection, treatment and storage to be a highly skilled activity which some nurseries have no interest in incorporating as part of their regular business practice.

**Fig. 1:**
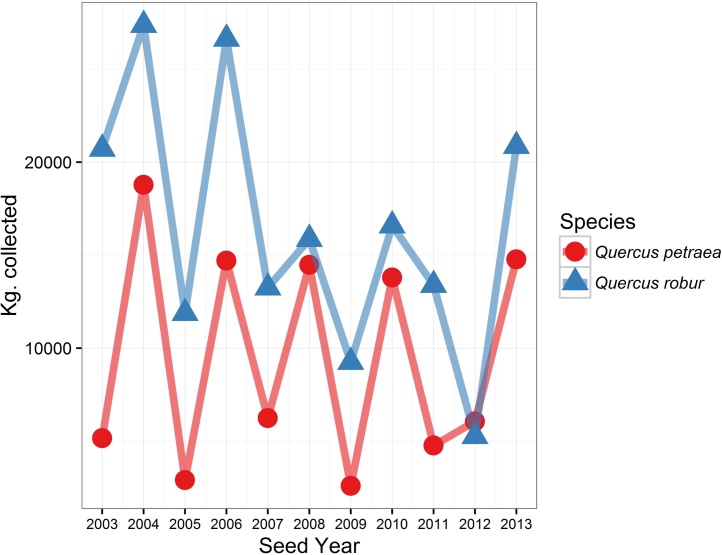
Annual quantity (kg.) of seed collected for the two native *Quercus* spp. in GB. Data obtained from the Forestry Commission FRM database.

**Fig. 2:**
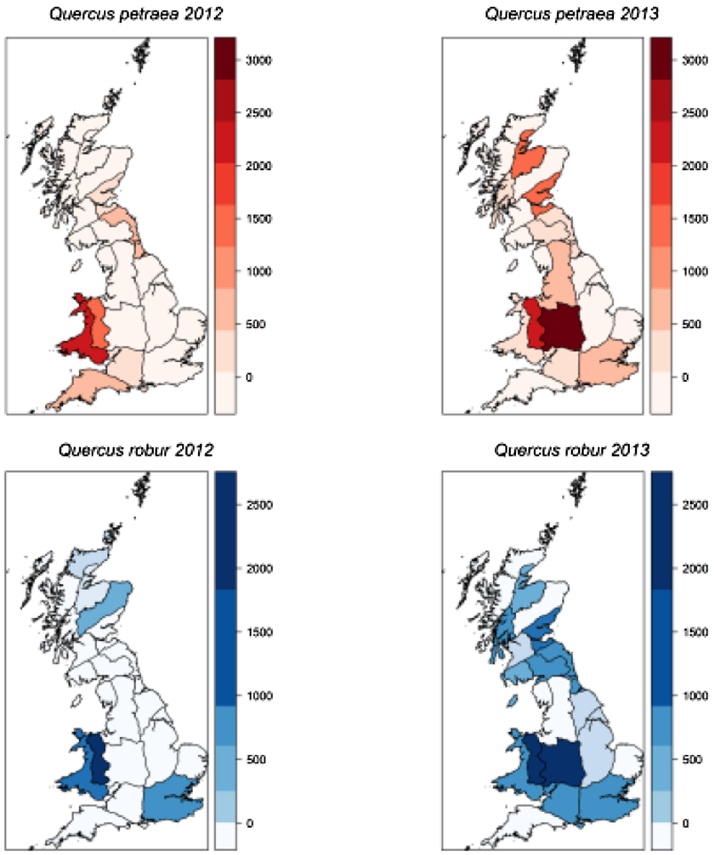
Location and quantity (kg.) of seed collected for the two native *Quercus* spp. in GB, in the years 2012 and 2013, summarised by seed zone. Breaks for colour coding indicate seed quantity for each species and were generated using the ‘sd’ style within the R package “classInt” (Bivand et al., 2013). The numerical scales are based on values for the year in which seed was more abundant—in both cases, 2013.

There can be considerable variation in availability of seed from year to year for some species. For example, oak trees (*Quercus* L. spp.), exhibit masting behaviour, with highly variable interannual seed crops (Fig. 1). In the case of oaks, this is further complicated by the seed being recalcitrant (dessication-intolerant) and cannot be viably stored for long periods (Gosling, 2007). In addition to temporal variability, most northern hemisphere trees exhibit spatially variable synchrony in seed production, meaning that, in some years, seed is produced in greater quantity in some places than in other places (Silvertown, 1980, Koenig and Knops, 2000). Spatial patterns of oak seed availability in GB differ between years (Fig. 2).

Variability in seed production is not always considered in woodland creation plans.

“*There hasn’t been a good acorn year in the last five years. It was good in the east last year* [2013] *but not here and we haven’t taken any orders yet the Forestry Commission are still approving schemes that are 60% planted oak, the customers are coming back and saying ‘what am I going to do?’ I can’t magic acorns out of nowhere*.” [S|P|N]

*“If nurseries don’t sell any oak then that means that they don’t sell any companion species either and all the schemes involving oak will be put off for a year.”* [M|P|N]

#### 3.2.2 The current system of seed zoning

Respondents were asked for their views of the existing system of seed zones (mapped in Fig. 2). Discussion tended to focus on two themes. Firstly, the biological relevance of the seed zones, i.e. whether adhering to local origin encourages the use of adapted material and secondly, the practical application of the seed zones for suppliers, i.e. whether seed zone stipulation helps or hinders their business operations.

One respondent, who had described difficulties with the seed zones, found it hard to envisage a viable alternative solution.

*“The lines on the map have to be there anyway to maintain bureaucracy. Creating separate zones for more species would create an even more convoluted system than is already present and it would become impossible to get what you want.”* [M|P|NEx]

Some were sceptical of the seed zone maps, calling into question their biological relevance.

*“I understand why we want seed zones and the reason for having them—climate et cetera but that does not adhere to how they are split at the moment. If you can split up the country using motorways and stuff like that then there isn’t much science behind it.”* [M|P|NEx]

*“I do feel that southern Britain is one outbreeding mass… I really have difficulty seeing much difference between 405,403,404* [three seed zones in the south of England]*.”* [M|P|N]

“*I think that you could get away with three zones in England*—*the semi-arid zone, the wet zone and the dry zone. Once you get north of the border* [Scotland]*, it’s a different story because topography plays a major role and I don’t think the current seed zone map allows for that*” [M|P|N]

Despite these doubts, there was some support for the seed zones, at least conceptually, as they add assurances to products, which gives the domestic sector a competitive advantage over producers elsewhere.

“*I’ve always thought that basically, if it hadn’t been for the seed zones, during the time of recession, there would be a whole lot less nurseries out there*” [M|P|N]

Almost all respondents commented that they experienced difficulties in supplying specific seed origins for a planting scheme at some time. The problems were linked to demand at short notice.

*“What I said at the time* [the seed zones were initiated] *is this is going to make our sales a complete lottery and if you’ve happened to grow the provenances that suddenly there’s a big planting scheme for then you’ve won the lottery but if you haven’t then there’s a load of stock which isn’t going to go anywhere.”* [M|P|Ex]

Respondents indicated that forest authorities in different parts of GB had differing opinions about the necessity to source local origin material. Several respondents mentioned that certain forest conservancies [administrative regions in which grants and guidance are issued] in Scotland and Wales were far stricter about seed origin and that at times this had hindered or even prevented initiation of planting schemes. English authorities were perceived as being more lenient regarding seed origin choice and were often content to accept non-local GB or non-GB material.

*“It depends which conservancy they are in. In Wales they like Wales, England is broader. Scotland is much stricter, especially in the Highlands.”* [M|P|N]

*“The Welsh office is always asking for Welsh provenance. Why is that? It’s not particularly botanical survival I think. It’s a political wheeze. What happens is you get all the landscape contractors going around all the nurseries asking for the right provenance spec. They might find five or six nurseries with a bucketful each of Welsh provenance, and where do they go? To some extent, the nurseries with the most flexible paperwork get the deal. I don’t know.”* [M|P|N]

Regional differences in provenance specification are due to differences in species behaviour in different environments. Local adaptation is likely to be much more frequent in heterogeneous landscapes with strong selective gradients (Kawecki and Ebert, 2004), such as upland regions in Scotland and Wales and so, arguably, it is logical to apply a more conservative approach in these areas.

Occasionally, using planting stock raised from seed collected in seed zones adjacent to a planting site is considered acceptable by the forestry authority (Forestry Commission Scotland, 2006). The influence that this flexibility has had upon the system was clearly recognised by one respondent, who had noticed that demand for seed of one region, 102 (northernmost Scotland) had reduced.

*“The situation is now that if you haven’t got the right zone, you’ve got to have the one next door to it. There’s nothing* [few other seed zones] *next to 102 so it’s not a very popular zone. If you go into 105 or 106, you’ve actually got 104, 102, 201 and 106. We haven’t got a clue what people want next year. We haven’t got a clue what people want tomorrow. So what you’ve now got is that the demand is coming from those provenances in which you can have the least amount of provenances but the most of the country covered.”* [L|P|N]

#### 3.2.3 Seed sourcing and climate change

Respondents were asked for their opinion of the practice of predictive provenancing, i.e. sourcing seed from areas which currently experience climatic conditions expected for planting sites into the future (Aitken and Whitlock, 2013, Breed et al., 2013, Prober et al., 2015). In a GB context, this would involve sourcing seed from locations 2–5 ° south of the planting site (Broadmeadow et al., 2005, Forestry Commission England, 2010, Morison et al., 2010, Weir, 2015).

Some felt that this would put the domestic trade at risk and have the unintended consequence of moving the market away from GB-grown material. Although most respondents were to some extent ambivalent, 12/16 of the respondents were either mostly sceptical or claimed that they did not understand the science but would not do it anyway. Two were very supportive of the concept and two felt that it did not matter. A common perspective was that indiscriminate sourcing of seed from currently warmer climates was not viable but that there was merit in the ideology of the approach as part of a general drive to diversify the base of material used.

Generally, respondents felt that climate change was more complicated than patterns of directional warming, and therefore sourcing seed from more southerly origins was not suitable as a single strategy.

*“Climate and weather are two different things. I believe in climate change but if we adapt to climate change, we also have to take account of the present weather conditions we are having. I think the ideology of thinking long term is correct but whatever we are thinking long term has got to be able to tackle the short term too.”* [L|P|N]

“*There’s only one thing I can guarantee you about the* [climate change predictions] *forecast, and that is that it is wrong, because all forecasts are wrong. We want to build in resilience for the unknown. If you’ve got known unknowns, don’t try and turn it into a known known because you never will.”* [M|P|N]

Some respondents gave very pragmatic answers.

*“We generally don’t go south because of the risk of frost damage.”* [S|T|Ex].

“*No* [sourcing seed from further south is not a sensible adaptation strategy], *because the local climate effects are huge*” [S|P|N].

Others were not convinced that it was necessary and that it may be best to spend more time considering the options.

*“I think that a foresters’ job is to manage his clients’ woodland. If there is a risk on the horizon, you consider it and have a think about how to mitigate or deal with it. Doing nothing can be all right though, as long as you have thought about it. It shouldn’t be chosen blindly. Trees do adapt, they can cope with a level of change.”* [M|P|NEx]

“*There has been too much action and not enough thinking. The issue with forestry is that foresters tend to be very proactive ‘do-stuff’ people but in this case it might be best to do absolutely nothing. People feel the need to do something although it’s not always necessary. There’s too much of “I want this to happen in my career”, but that shouldn’t be the case.*” [M|P|N]

Another response was that it would make little difference, due to widespread historical imports of plant material.

*“I would think that there is such a vast amount coming in from Holland, Belgium and France that the mix of crop already in the UK always has been coming from those areas and that it wouldn’t make much difference.”* [L|T|Ex].

Supplying alternative products was recognised as a niche marketing opportunity.

“[There are] *some people who are living off the back of these recommendations and making money from it. I’m not sure they necessarily agree with it but it is a marketing opportunity*” [M|P|N]

One respondent was very supportive of the move for southerly origins.

*“Yes. In the right territories, within reason, assuming it’s all ok and disease free and not bringing in anything different up into the UK. Yes, definitely, yes. That’s what we’re doing, that’s what we’re getting customers asking us for. I think the people who are looking for climate change tolerance or testing out these species are people who are more serious productive people. Growing people. Thinking people.”* [M|P|NEx]

### 3.3 Plant supply

#### 3.3.1 Grant schemes

Forest planting schemes in GB tend to rely on subsidy support from grant schemes funded by the UK’s share of EU Common Agricultural Policy funds. Beginning in 1988 with the Woodland Grant Scheme, there have been six grant schemes in Scotland and five grant schemes in both England and Wales, with an average duration of 4.8 years. Additionally, subsidy rates for different activities and policies vary both between and within grant schemes at times – and the administrative systems required for their implementation are revised, which can create delays (Macaskill, 2016). Respondents to the survey were asked to comment on problems they have experienced with grant schemes and for their opinions regarding possible changes to grant schemes which might improve the efficiency of the plant and seed supply chain.

“*We should have a system reflecting that the industry is long term and not moving the goal posts every five years*. *If you remove the politics of it, you get an overarching strategy in place for twenty years that is the best thing for the sector*” [L|P|Ex]

**Fig. 3:**
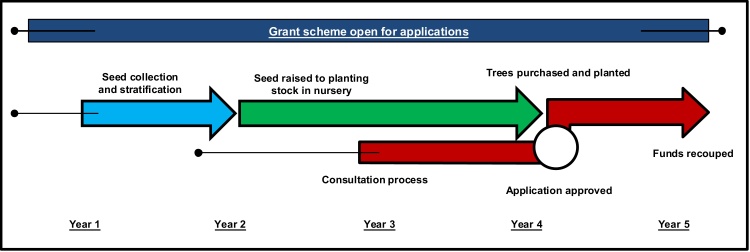
Idealised timeline diagram of events involved in plant production and grant application and approval. NB there is likely to be much variation in the time any one of these activities may take. This variation is likely to be particularly strong for seed collection and stratification, grant approval and grant scheme duration. This is indicated by the additional narrow tails.

Due to grant stipulations, and the long period of time it can take to secure funding, forest managers usually provide nurseries with specifications with little notice − despite nurseries requiring up to three years to produce a tree seedling which is ready to be deployed to the planting site, and longer if targeted seed collection is required. If grant application took place before plant specification, the entire process would be likely take longer than the period in which a single grant scheme is open for (Fig. 3).

“*Most of our clients are coming to us and saying, I would like to buy one million plants. When do they need them? Two weeks. They are all purchasing plants for the season we are already in or the season we are about to enter. That’s to do with the amount of time it takes for grant approvals to go through*.” [M|P|NEx]

“*You take a forester who is specifying to his or her nursery two weeks before they want it delivered. They want a certain species, of a given size, of the correct provenance. They’re also now specifying where it is grown, of a given altitude and want it in two weeks’ time. How is the nursery trade supposed to produce this product?*” [L|P|N]

This view was extended by some respondents, who suggested that subsidy schemes were not conducive to long term management.

“*If you want the really big answer, you would remove all of the agricultural and forestry grants. The blackface sheep would come off the hillsides and the price of land would come down and after a few years people would really be thinking about what they want to achieve by doing x, y or z on that hill. There is no room for people to sit down with the client and say – ‘what do you want for your estate? What is it going to look like in 30 years? What do you want to achieve?*” [S|P|N]

“*I personally, would like to see the industry move away from direct support. I think it would come out stronger*” [L|P|N]

“*We shouldn’t have taken away the tax concessions* [of the 1980’s]*. The people who were getting them weren’t taking money; it just meant that the tax was deferred. It was a good system. A company I used to work for were sending out lorry loads of trees and when that ended it just stopped*” [S|T|Ex]

#### 3.3.2 Trading in live plants

Due to the prevalence of speculative production in GB nurseries, trading among nurseries to fulfil stock requests is common. In addition to trading amongst GB nurseries, planting stock is also sourced from large scale nursery enterprises in other countries, especially in Western Europe (Russell and Evans, 2003). In our survey four out of the sixteen respondents did not import any planting stock from other countries. Those that did import planting stock indicated that they generally did so because they could rely on the quality of products and services and trusted their trading partners.

*“The producers in Europe grow excellent stock. It is a safety net for us. We produce what we know we can sell. If for any reason, there is an increase in demand, we can meet that by importing”* [M|P|NEx]

*“When the ash dieback thing* [outbreak of *Hymenoscyphus fraxineus* (T. Kowalski) Baral, Queloz, Hosoya infection of ash trees] *happened, people were saying, why were you and the nursery trade importing such vast amounts of ash from abroad? I suppose it was spontaneous demand and unusual specifications late in the season. This spontaneity doesn’t help stability in British production.”* [M|P|N]

One trading nursery that relies entirely on hedging or forestry-purposes stock grown outside of GB had tried but failed to commit to exclusively supplying GB-grown planting stock.

*“We decided last year that what we would try to do was buy British. And so we started buying more in this country but they weren’t able to do what we wanted them to do. We managed it for about two months, completely hit and miss deliveries and they were delivering the wrong size. It was complete chaos and so we went back to what we were doing before, sadly, buying from the continent.”* [S|T|Ex]

“*Often the choice is between having continental seed grown here or British seed grown on the continent. So you can have an imported plant of the right provenance or the wrong provenance that is grown in the UK.*” [M|P|NEx]

Purchasing and selling trees grown elsewhere can be profitable, and negates some of the risk associated with speculative production.

*“We work pretty closely with two other* [GB] *nurseries and these are people I know I can trust. I can make money from selling other people’s trees. If we just sold our own trees, we’d be pretty poor.”* [S|P|N]

Two respondents suggested that there are ways to bypass the marketing certification system and that, at times, European suppliers have taken advantage of weak policing of the FRM system by supplying false documentation.

*“I work widely in the European market and some of the things I am asked to do are blatant fraud. They’re looking for someone to produce the paperwork − that goes on widely.”* [L|P|N]

*“They* [overseas suppliers] *will say – you don’t need the certificate, just tell your customer lies. I think by and large we do get it right in this country but I think we need to be slightly more aware that not everybody is honest and truthful.”* [M|P|N]

**Fig. 4:**
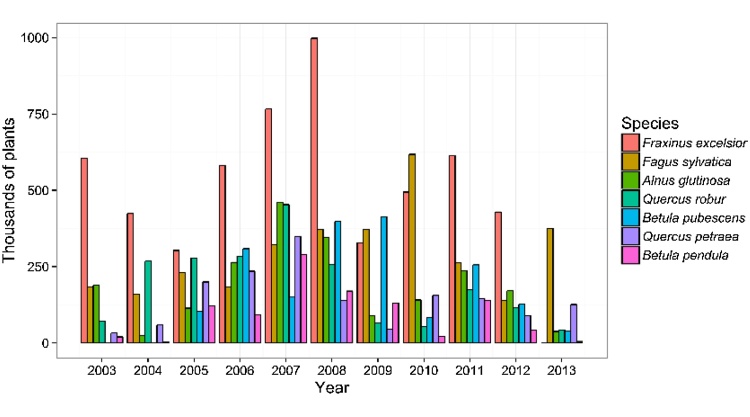
The number of plants (thousands) recorded as being imported to GB 2003–2013 for species in which the total number of trees imported exceeded one million, according to the FC FRM database.

**Fig. 5:**
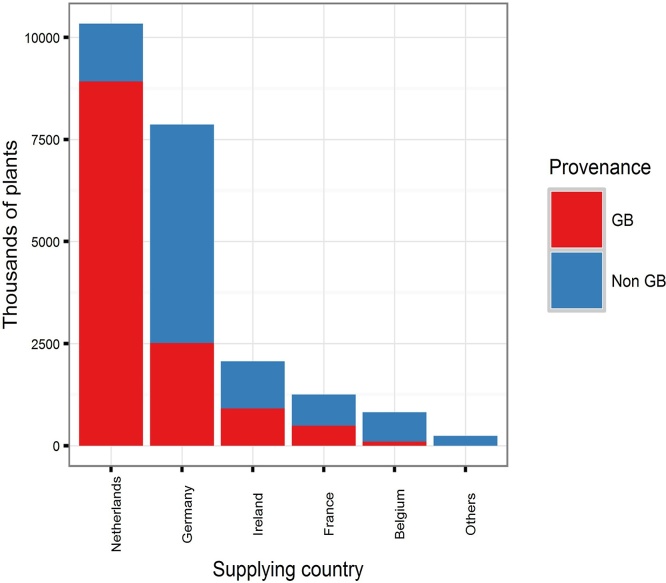
The supplying country of imported planting stock presented in Fig. 4. This is broken down by country of provenance (GB/non-GB), to demonstrate the proportion of imported planting stock which is of GB provenance.

The Forestry Commission FRM database records the number of plants imported for regulated species for which they have been notified. These can be broken down by year and by species (Fig. 4). In total, approximately 59% of plants recorded as imported for 2003–2013 were certified as being of GB provenance (i.e. raised from seed collected in GB), but supplied by other countries (Fig. 5). The CONFOR nursery producer’s group which, at the time comprised seven of the largest forest nurseries in GB, estimated that their members imported at least 10 million plants in 2012 (Anon, 2012). Fewer than half of this number (36.5%) appears in the FC FRM database for that year, suggesting that the estimates we derived from the national databases are likely to be lower than the actual number of imported plants. In any case, the proportion of imported versus non-imported trees, which is estimated as 12.5% in Anon (2012) is much lower than the 70% estimated for native broadleaves in 1993 (Gordon, 1998). This is in line with recent trends in customer preference for GB-grown material. A recent survey identified that 69% of woodland owners stated a preference for GB-grown trees for the future (Hemery et al., 2015). Interestingly, the intention to specify particular provenances is predicted to decline. A small majority of 54% of survey respondents claiming that they have tended to specify provenance in the past but only 44% claim that they will continue to do so into the future (Hemery et al., 2015). This suggests that less value will be placed on provenance than the location of supplier by forest owners into the future, contrasting with trends in the past decade in which importation of GB provenance material has been widespread (Fig. 5).

**Box 1 Uncertainty begets importation**

Using quantitative data offered by one respondent, we aim to present an example of problems which can be caused by rapid shifts in policy and subsidy support.

**Fig. 6:**
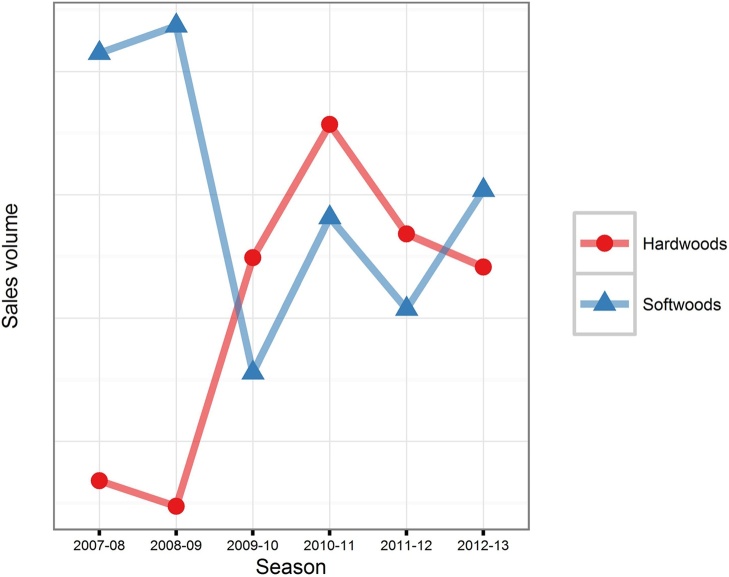
Annual sales volume for softwoods and hardwoods at a private sector nursery 2007–2013.

“*Overnight the demand shifted because the grant rate was more attractive for hardwoods in the new scheme* [(Fig. 6)]*. We can’t magic plants out of thin air, so when the demand for softwoods dropped* – *the proportion we still have on the nursery gets burnt because we can’t sell it*”

In this scenario, there was a rapid shift from one subsidy scheme to another in 2007. In the latter scheme, more attractive rates of subsidy were available for broadleaved species (especially agricultural hedging) than before. This influenced demand at very short notice to the nurseries – and as such, conifer crops which were already being grown at this nursery were destroyed as subsidy rates were less competitive. The nursery was able to diversify quickly by importing planting stock.

“*There was a hedging grant. That allowed us to survive the transition because of that hedging. We were able to import those plants because provenance wasn’t important – the farmers didn’t care about the provenance of their hedges. That increase buffered that decrease* [in conifer sales] *which is why we are still here*”

**Fig. 7:**
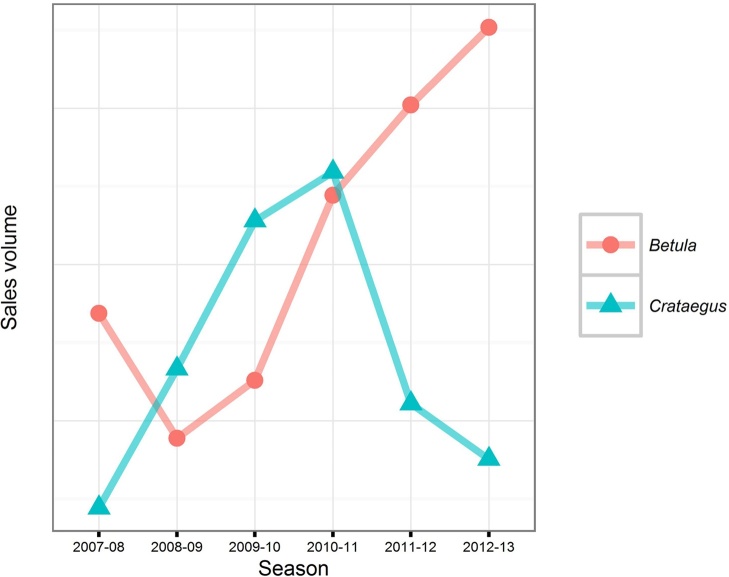
Annual sales volume for *Betula* spp. and *Crataegus monogyna* at the same nursery between 2007 and 2013. Note—There are no values on the Y axes, as these data are confidential. Additionally, the scale of the Y axes of the two plots (Figs. 6, 7) is not equivalent − these data are used to indicate magnitude and thus should be interpreted qualitatively.

However, in 2010 the subsidy rate for hedging was removed, again, at short notice, and without prior consultation with the nursery sector (Fig. 7), but. in this case, reduced *Crataegus monogyna* Jacq. sales were buffered increased in *Betula* L. spp sales.

“*If I had decided here* [3 years prior] *– the market looks good for this* [hedging] *so I will sow loads of them, I would have been burning them at this point. So that ability to import is in my opinion necessary until the market is stable enough to allow advance purchase of plants*.*”*

**Fig. 8:**
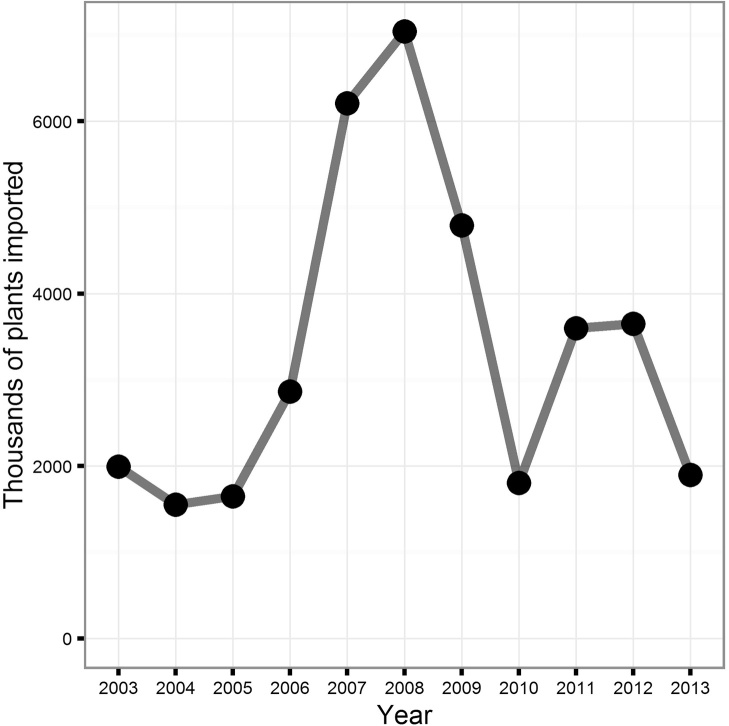
The total number of plants (thousands) recorded as being imported to GB 2003–2013 for all species, according to the FC FRM database.

Looking more widely, there seems to be a relationship between the volume of imports and transitions between grant schemes. The change from the Scottish Forestry Grant Scheme to the Scottish Rural Development Programme occurred between September 2006 and January 2007. There were major revisions to the English Woodland Grant Scheme between 2007 and 2009 and a new grant scheme in Wales, “Better Woodlands for Wales”, opened in September 2006. The period between 2006 and 2009 is when the highest number of plants was reported as having been imported (Fig. 8). It seems likely that these two factors are related and supports the claims made by the nursery used in our example of grant scheme transitions.

#### 3.3.3 Advance purchase of plants—contract growing

Contract grows, whereby a customer specifies a particular seed origin, either by providing the seed or contracting a collection in addition to growing the plants are one option which may add assurance to crop production. The advantage of contract growing is that the stock can be grown in addition to normal production, with an agreement on the sale in place at the time of sowing. This is common practice in some countries, e.g. Finland (Rikala, 2000) and the United States (Haase, D. *personal communication*), especially for large planting schemes.

Few examples of contract growing were found within the domestic sector in GB. One nursery reported that 60% of their stock was grown under contract and another reported 40%. For all of the other respondents, the proportion was lower than 5% and several said that it had happened once or twice or never at all.

Generally, nurseries were amenable to the idea of advance purchase of plants, although several respondents made it clear that contract growing is not a *panacea* and it does present its own difficulties:

*“Yes, it has its difficulties but if people want a particular seed origin and if it’s seed from ancient woodland or something special then it is definitely a good idea.”* [S|T|Ex]

One respondent noted that contract growing is a partial solution, but could not replace speculative production.

*“On spec, we don’t know who is going to take it* [planting stock] *but we know that somebody will. Even if half of that were on contract, it wouldn’t make the slightest bit of difference to the other half. That would still be speculative. Contract grows are a bit of a red herring.”* [M|P|N]

For smaller producers, entering into a contract to supply plants is often more of a risk than speculative production.

*“No contract grows, it is too much risk. Too much risk for ourselves, contract prices are low prices and if we have a disaster it’s a big disaster, then you have to go out and re-buy the stock.”* [S|P|N]

## 4 Discussion

### 4.1 Tree seed sourcing

The availability of tree seed is the first limiting factor in any seed sourcing process and is subject to the vagaries of nature, especially when harvested in field conditions (Broadhurst et al., 2015). Strategies to improve availability could either involve increasing collection effort *in situ*, increasing seed production *ex situ* (Broadhurst et al., 2016) or investigating technology to increase the period of time for which germplasm can be stored without losing the ability to germinate (Gosling, 2007). In the immediate absence of these capabilities, organisations responsible for overseeing grant applications should make better recognition of these natural fluctuations in availability of seed. Whilst this may entail delays in planting, it is preferable to deploying planting stock of an inappropriate seed origin or species for the planting site and preferable to importing the planting stock (Hubert and Cundall, 2006). Grant schemes do not currently offer enough flexibility to allow for this, as they place a time limit on completion of works following approval.

The current system of seed origin choice (seed zones), clearly creates problems for suppliers, as they increase the number of product lines a supplier is expected to manage beyond those which would enable a nursery to produce any specific seed origin in volume. This is a problem because tree seedlings are perishable and must be sown long before they are available for sale, a process which typically takes 1–3 years. Without prior knowledge of demand, nurseries must be judicious when sourcing seed and decide whether to grow large quantities of trees from few seed origins (high risk, high reward), or smaller quantities of trees from many seed origins (lower risk, lower reward). Of course, this risk is a reality for any commercial enterprise but it can lead to negative consequences for genetic resource management (inappropriate material planted), biosecurity (excessive reliance on imports) and leads to unnecessary waste. It is important to remember that much of the capital supporting this industry is indirectly derived from taxpayers on the understanding that woodland expansion delivers public benefits.

A survey in British Colombia and Alberta discovered that support for reforestation with non-local seed for climate change adaptation amongst the general public was around 60%, and that increasing levels of knowledge of reforestation technology increased the likelihood of acceptance of the strategy (Hajjar and Kozak, 2015). Our sample was comprised of highly knowledgeable individuals and although it is smaller than necessary to make statistical inference, the GB nursery sector seems to be more sceptical about predictive provenancing. This may be due to the much smaller scale, and perhaps inherently more commercially conservative status of the forest industry in GB than in Canada. Nonetheless, most of the criticisms of predictive provenancing (sourcing seed from more currently warmer locations) were related to the biological considerations (Section 3.2.3).

In addition to biological considerations, some practical and economic problems with predictive provenancing emerged from the survey. Landholders of seed stands typically receive a proportion of profit achieved on the sale of seed collected from their woodlands. A shift to non-local seed origins could remove the incentive for landholders to manage or allow access to seed stands or increase costs of obtaining seed if collectors are required to source seed from further afield. This would increase the wholesale cost of seed and the costs of woodland establishment. If nurseries are required to speculatively produce planting stock from additional seed origins to those already grown, this will add further risk to their own investments than already exists. Finally, if the suggested practice of mixing the seed origins of planting stock at a single planting site is adopted (Forestry Commission, 2011), a likely scenario, given the existing difficulties associated with predicting demand, is that managers will either have to accept whichever seed origins a nursery sows or accept that trading will be required to provide planting stock of multiple seed origins simultaneously.

More research is required to identify major patterns of adaptive variation in GB tree species, and this should inform policies related to seed sourcing (Boshier and Stewart, 2005, Cavers and Cottrell, 2015; Whittet et al., in press). For instance, several respondents perceived that there are currently more seed zones than is necessary in the relatively homogeneous south of England, as has been demonstrated for Black alder *Alnus glutinosa* L. Gaertn. in Belgium (De Kort et al., 2014). In this case, small seed zones may make seed collection and stock control more complicated than necessary, with no obvious fitness advantage of using local material (Hubert and Cottrell, 2007, O’Neill et al., 2014). A more bespoke, biologically relevant system for sourcing currently adapted seed would not necessarily be more restrictive or complicated than that the current system of seed zones. Such a bespoke system, based on scientific evidence, would also have the advantage of better predicting tree survival at planting sites. This coupled with a greater capacity to access documented and stored seed would add security to the supply chain.

### 4.2 Plant supply

Demand or at least a preference for GB-grown planting stock is increasing, partly brought about by awareness of plant health problems (Hemery et al., 2015). This greater emphasis on home-grown planting stock should theoretically improve the competitiveness of the domestic nursery sector. However, whilst uncertainty created by the configuration of GB grant schemes remains, there is little indication that imports are likely to cease in the foreseeable future. Large nurseries in mainland Europe have the volume, infrastructure and climate to produce a greater number of product lines, including those grown from GB provenance seed or traded in from elsewhere in Europe. These efficiencies of scale provide continental producers with the confidence to grow trees from a range of GB seed origins speculatively and still make sustainable profit margins by selling back into GB or elsewhere.

Contract growing, in the strict sense, is not an ideal solution to the problem of unpredictable demand, since it requires the supplier and customer to enter into a legally binding agreement, which itself is not free from risk. Contract grows are useful when stock specifications are very tight or when the product being sought is not typically carried by a supplier, especially if targeted seed collection is required. However, in some instances they are unattractive because the sale price may be lower. If nurseries are tied completely into contracts, they will lose the ability to innovate, or gain higher rewards associated with speculative production. In any case, contract growing relies on consumer confidence, which is currently lacking and is a major bottleneck in sustainable seed and plant supply.

Transitioning from a subsidised to a free-market status was mentioned by some of the respondents as a way of increasing consumer autonomy and confidence. Decoupling from agricultural subsidies took place in New Zealand in the 1970′s. This was initially followed by a steep decline in the country’s agricultural human population and subsequently led to intensification of the agriculture sector (MacLeod and Moller, 2006). Effects of liberalising the market in GB would be complex and would constitute very radical reform (Potter, 1996). A possible scenario is that it would lead to reluctance to deliberately create non-profit making native woodlands by private landholders. On the other hand, reducing the rates of subsidy, or adopting a more moderate cost-sharing incentive scheme rather than direct support might entail a shift away from native woodland creation by *materialistic/profit-seeking* landholders to *recreational* landholders (*sensu* Serbruyns and Luyssaert, 2006), meaning that deliberate woodland creation for non-financial purposes would be conducted only by those who are genuinely interested in and motivated by positive environmental outcomes. Another indirect consequence of removing agricultural subsidy might be natural colonisation of formerly agricultural land by trees in instances where currently subsidised activities become economically inviable without financial support, particularly in remote areas less favoured by intensive agriculture (Potter and Goodwin, 1998).

Although in-depth analysis of alternative modes of incentivising native woodland creation and expansion is beyond the scope of this paper; the most obvious consensus from respondents is that more stable grant schemes would allow nurseries to operate efficiently and plan over much longer time-scales than they are currently able to do. Greater flexibility and tolerance of changes to individual planting schemes where there are legitimate reasons for doing so (e.g. inability to procure GB-grown plants of appropriate origin) are also a priority.

To achieve this, it is necessary to develop simpler and more reliable administrative systems for processing grant proposals. Decentralisation of some aspects of the approval process may also enable more efficient delivery of woodland creation and expansion projects whilst taking advantage of local knowledge. Of course, over time, it may be necessary to modify some guidance and policies as our understanding of environmental change develops and to reflect naturally changing requirements of the industry. However, any such changes must be coupled with extensive consultation between the public and private sectors and notice should be provided long in advance of changes, especially to nursery producers in the private sector. Nursery producers are arguably exposed to the highest level of the risk in the supply chain (Broadhurst et al., 2016), despite the fact that their ability to produce and compete with suppliers elsewhere to supply plants for native woodland expansion is clearly in the public interest.

## 5 Conclusions

The ability to create resilient and healthy woodlands from nursery raised planting stock depends on the existence of a resilient domestic seed and plant supply chain to support these efforts. Identifying challenges faced by the forest nursery sector in Great Britain has revealed that bottlenecks in the supply chain are principally natural (seed availability at a given time) and bureaucratic (grants and regulations). Little can be done to mitigate the former bottleneck in the short term. However; greater tolerance at an administrative level may go some way towards easing the constraints it poses. Across the sector currently, productivity and competitiveness are hindered, not by an intrinsic lack of capacity in the GB domestic forest nursery sector, but by a lack of long term market predictability which leads to overproduction and waste on one hand and underproduction and consequent reliance on imports on the other. This analysis suggests that better scientific information to guide seed sourcing − and the tools to use it efficiently − is required to guide seed sourcing policies under uncertain future conditions. Sourcing appropriate planting stock is an inherently long term process and so for such information to be adopted operationally, much more stable and efficient administrative systems for financing and regulating native woodland creation than currently exist are required. In the absence of consistent policy, it may be necessary to revisit stated planting targets and ask whether these are achievable, and at what costs? Finally, an important conclusion from this paper is that it is crucial for scientists and policy makers to consult with industry to determine the practicability and economic viability of any change to forest policy.

## Funding

This work was supported via a studentship funded by Forestry Commission Scotland; Forestry Commission GB and ClimateXChange. Further support was provided via PROTREE ‘Promoting resilience of UK tree species to novel pests and pathogens: ecological and evolutionary solutions’, a project funded by the BBSRC Tree Health and Plant Biosecurity Initiative, Phase 2, an initiative of the Living with Environmental Change (LWEC) partnership.

## Figures and Tables

**Table 1:** Characteristics and codes of the survey respondents.

Attribute	Nursery size			Trading status		Majority market (species)		
Category	Large	Medium	Small	Producer	Trader	Natives	Exotics	Equal
Number	4	7	5	12	4	7	6	3
Code	L	M	S	P	T	N	Ex	NEx
