# Supplementary material for: Supplying trees in an era of environmental uncertainty: Identifying challenges faced by the forest nursery sector in Great Britain
Source: Land use policy. 2016 Dec 15;58:415–26. doi: 10.1016/j.landusepol.2016.07.027 (PMC5045158; doi:10.1016/j.landusepol.2016.07.027)
Supplement: Supplementary file 1 [file mmc1.docx]

**S1. Supporting information – nursery interview guideline.**

*This is a list of questions used as an interview guideline in the qualitative surveys conducted with forest nurseries and seed merchants in 2014 and 2015. Please note that this list of questions is a guideline only. Many of these questions are open-ended and did not receive discrete responses. Some questions (e.g. those related to seed collection), were not relevant to every respondent. Also, some questions were not answered by some respondents.*

1. How many full time employees does your organisation have?
2. How many seasonal workers would you employ in a normal year?
3. Roughly, what proportion of customers are end users? What about contractors or trade nurseries?
4. What proportion of your client base is located in GB?
5. How far away most of the trees you supply planted out? Does your customer base have a core region?
6. What proportion of the orders for native tree species you supply is contract grown?
7. What proportion of turnover does this represent?
8. Would you like to see this proportion increasing and do you think that changes to the grant system are needed to allow this?
9. Are you bare root producers or cell/container growers? If both, then roughly what proportion?
10. How many trees do you produce in a normal year?
11. What proportion of your turnover is generated by trade in native tree species; 1-10?
12. Which native tree species have you made your own collections of in the last three years? Which seed zones, or stands?
13. Briefly, how is this organised?
14. Which of these species have you imported in the last three years? Or attained through another party who imported seeds/parts of plants? Which countries of provenance?
15. How many individuals are involved in your collection network?
16. Do they supply you exclusively?
17. Where do you send your seed for testing?
18. What, if anything, would discourage you from collecting local seed? Are some species particularly difficult and what are the problems associated with collecting them?
19. If you collect surplus seed, what happens to it?
20. Do you have seed storage facilities?
21. When you have to buy in seed, where do you get it from?
22. What do you do when you cannot supply what clients ask for? Are you in a position to recommend alternatives or are they usually too limited by grant schemes?
23. What proportions of the orders you supply can only be achieved by partially outsourcing to other organisations?
24. What proportion of your client base use subsidies? How does this vary by sector (private commercial forestry; environmental; public sector; utilities)?
25. What do you think are the customers’ main priorities, i.e. what sort of specifications do you typically receive and are customers exacting about provenance choice? Are some less interested?
26. Do specifications vary by sector?
27. Does the current system of seed zoning used in GB promote the use of adapted planting stock?
28. Are you aware of any problems in the establishment of trees being planted in their local seed zone?
29. Do customers ever mention climate change in their specifications? – Or attempt to source specifically for climate change adaptation?
30. What changes are necessary in the grant system to promote closer-to-ideal practice?
31. What can you see changing in the system?
32. Are there things you would like to be doing which you can’t necessarily do because of lack of demand/poor return?
33. What would be required to change this?
34. Is sourcing Southern provenances an appropriate strategy for climate change adaptation?
35. Do you expect the proposed new FRM regulations to have an impact upon your business?
